# Complement C1q Enhances Primary Hemostasis

**DOI:** 10.3389/fimmu.2020.01522

**Published:** 2020-07-16

**Authors:** Claudia Donat, Robert Kölm, Kinga Csorba, Eylul Tuncer, Dimitrios A. Tsakiris, Marten Trendelenburg

**Affiliations:** ^1^Laboratory of Clinical Immunology, Department of Biomedicine, University of Basel, Basel, Switzerland; ^2^Department of Diagnostic Hematology, University Hospital Basel, Basel, Switzerland; ^3^Division of Internal Medicine, University Hospital Basel, Basel, Switzerland

**Keywords:** complement system, complement C1q, hemostasis, von Willebrand factor, coagulation, thrombosis, bleeding time

## Abstract

The cross-talk between the inflammatory complement system and hemostasis is becoming increasingly recognized. The interaction between complement C1q, initiation molecule of the classical pathway, and von Willebrand factor (vWF), initiator molecule of primary hemostasis, has been shown to induce platelet rolling and adhesion *in vitro*. As vWF disorders result in prolonged bleeding, a lack of C1q as binding partner for vWF might also lead to an impaired hemostasis. Therefore, this study aimed to investigate the *in vivo* relevance of C1q-dependent binding of vWF in hemostasis. For this purpose, we analyzed parameters of primary and secondary hemostasis and performed bleeding experiments in wild type (WT) and C1q-deficient (*C1qa*^−/−^) mice, with reconstitution experiments of C1q in the latter. Bleeding tendency was examined by quantification of bleeding time and blood loss. First, we found that complete blood counts and plasma vWF levels do not differ between *C1qa*^−/−^ mice and WT mice. Moreover, platelet aggregation tests indicated that the platelets of both strains of mice are functional. Second, while the prothrombin time was comparable between both groups, the activated partial thromboplastin time was shorter in *C1qa*^−/−^ mice. In contrast, tail bleeding times of *C1qa*^−/−^ mice were prolonged accompanied by an increased blood loss. Upon reconstitution of *C1qa*^−/−^ mice with C1q, parameters of increased bleeding could be reversed. In conclusion, our data indicate that C1q, a molecule of the first-line of immune defense, actively participates in primary hemostasis by promoting arrest of bleeding. This observation might be of relevance for the understanding of thromboembolic complications in inflammatory disorders, where excess of C1q deposition is observed.

## Introduction

Numerous interactions between the complement and the coagulation cascades have been described over the years. Since both pathways are thought to have evolved from a common ancestor, it is not surprising that structural as well as functional similarities exist between them (1). Structurally, both pathways are composed of potent serine proteases, which are circulating as inactive zymogens. Functionally, both systems belong to the first-line of defense and are intended to act locally at the site of infection/injury in order to limit collateral damage.

The complement system can be activated through the classical, lectin and the alternative pathways, with all three pathways leading to a shared effector response characterized by the formation of C3 and C5 convertases, release of the effector molecules C3a and C5a, and assembly of the membrane-attack complex.

The coagulation system can be characterized by the interaction between primary and secondary hemostasis. During primary hemostasis upon tissue damage, a concerted interplay of von Willebrand factor (vWF), collagen and platelets results in adhesion of platelets at the site of injury. Subsequent platelet activation and aggregation leads to the formation of a primary, instable platelet clot. During secondary hemostasis, exposure of blood to tissue factor (TF) initiates binding of factor VII, which in turn becomes activated. This leads to downstream activation of factors IX and X, with factor Xa being able to convert prothrombin to thrombin. Thrombin can amplify the cascade by activating factors XI, V, and VIII, as well as platelets themselves. Notably, to enable factor VIII to reach the phospholipid surface of those platelets, vWF is required as a carrier protein. The formation of a factor Xa-Va (prothrombinase) complex propagates the additional generation of thrombin, which then cleaves fibrinogen into insoluble fibrin. Cross-linking of fibrin polymers by factor XIIIa transforms the initial platelet clot into a stable clot. The current view is that primary and secondary hemostasis act in synergy rather than one after the other (2).

To date, experimental studies have described a functional impact of complement components on coagulation (3, 4). Activation of complement is commonly known to activate hemostasis, but there are also functions of complement components that are independent of complement activation as described for C1q–the pattern-recognition molecule of the classical complement pathway. On the one hand, C1q has been described to interact with platelets but data on this interaction are conflicting. While some studies demonstrate that C1q enhances platelet activation and upregulates P-selectin expression (5, 6), other studies rather suggest that C1q mitigates coagulation by inhibiting collagen-induced platelet aggregation (7, 8). On the other hand, C1q has been described to interact with factor XII, hereby proposing an inhibitory effect on clot formation (9). Additionally, the interaction of C1q with negatively charged molecules such as heparin derivatives (10, 11) or chondroitin sulfate A (12, 13) has been described to limit complement activation but to also activate platelets. Moreover, previous findings of our group have demonstrated that a complex of surface-bound C1q and vWF is able to induce platelet rolling and adhesion (14). This observation is of importance as activated components of complement, including C1q, and coagulation are frequently encountered concomitantly in thrombotic complications that accompany inflammatory disorders such as bacterial sepsis and systemic lupus erythematosus (SLE) (15–17).

Therefore, this study aimed to investigate the *in vivo* relevance of C1q-mediated binding of vWF by studying C1q-deficient mice with regard to alterations in hemostasis.

## Materials and Methods

### Animals

C57BL/6 mice (animal facility of the Department of Biomedicine, Basel, Switzerland) and *C1qa*^−/−^ mice on a C57BL/6 genetic background were maintained in our specific-pathogen-free facility at 22 °C room temperature (RT) with 12 h light/12 h dark cycle and were housed in groups of 2–6 mice. Mice used for experiments were kept for 2 weeks of adaptation period upon transfer. All procedures were approved by the Cantonal Commission for Animal Experiments, and the Federal Food Safety and Veterinary Office (license number 2898/28447). This study was carried out by authorized staff in accordance with the guidelines and regulations of the Swiss welfare legislation (Animal Welfare Ordinance, Animal Welfare Act and the Animal Experimentation Ordinance).

### Tail Bleeding Time and Blood Loss

For these experiments, 6–14 week old wild type (WT) or *C1qa*^−/−^ mice were weighed and injected with a mixture of ketamine (100 mg/kg body weight (BW), xylazine (10 mg/kg BW) and atropine (1.2 mg/kg BW) before 10 mm of the distal tail was amputated and immersed into 0.16% EDTA/PBS, kept at 37°C. Time to cessation of blood flow was evaluated for 15 min. Blood loss was analyzed by (i) reduction in body weight, calculated by reweighing the animals including amputated tails before and after tail bleeding, (ii) reduction in body weight normalized to total body weight, and (iii) optical density (OD) of blood-PBS solution obtained from tail bleeding assay. OD was analyzed in 96 well-plate using a microplate reader with an emission wavelength of 550 nm. Experimental set-up is shown in Supplementary Figure 1. In all these experiments, the experimenter was blinded to the genetic background of the animals or the substance of reconstitution.

### Complete Blood Counts

Whole blood was obtained from the tail vein, anticoagulated with EDTA and diluted with 0.9% saline (1:3). Blood cell counts and hemoglobin concentration were analyzed by using the ADVIA 2120i Hematology System (Siemens Healthcare, Erlangen, Germany).

### vWF Plasma Levels

Citrated plasma was obtained from the tail vein and analyzed for vWF levels using von Willebrand factor two matched antibody pair kit (Abcam), according to the manufacturer's instructions.

### Prothrombin Time (PT)

Citrated whole blood was obtained from the tail vein and analyzed using Hemochron PT citrate cuvettes and Hemochron Jr. Signature+ (both from Accriva Diagnostics, Instrumentation Laboratory, Bedford, MA, USA).

### Activated Partial Thromboplastin Time (aPTT)

Citrated plasma was obtained by cardiac puncture and analyzed for aPTT using ACL Top 750 Las (Instrumentation Laboratory).

### Platelet Function

Whole blood was obtained retro-orbitally, anticoagulated with hirudin and diluted with 0.9% saline (1:1) and analyzed for whole-blood platelet aggregation using the multiple electrode platelet aggregometry (MEA) Multiplate® Analyzer (Roche Diagnostics, Basel, Switzerland). Aggregation (electrical impedance) was induced with the platelet activating agonists ADP (15 μM) or collagen (10 μg/ml) and recorded for 6 min. In order to achieve sufficient amounts of platelets, blood was pooled from four mice per group.

### C1q Levels and Reconstitution of C1q

To determine kinetics of C1q reconstitution, *C1qa*^−/−^ mice were injected intraperitoneally (*i.p*.) with 500 μg purified human C1q (1 mg/ml, Complement Technology) and blood sampling from the tail vein was carried out at 30 min, 1, 2, and 8 h. Data are shown in Supplementary Figure 2. For the comparison of aPTT, platelet aggregation, bleeding diathesis and lectin pathway activity with or without reconstitution, mice were injected *i.p*. with 500 μg purified human C1q or the same volume of 0.9% saline 2 h prior to the experiment. The degree of reconstitution with C1q was quantified from the obtained serum (time point experiments and lectin pathway activity) or plasma (aPTT and tail bleeding experiments) using a mouse C1q ELISA kit (Hycult Biotech, Unden, Netherlands) with cross-reactivity to human C1q according to the manufacturer's instructions. The cross-reactivity of this ELISA kit for human C1q was exploited to analyze concentrations of mouse and human C1q. Since human C1q gave only approximately one third of the signal of the kit's mouse C1q, our achieved C1q plasma concentrations should be regarded as minimum concentrations. Despite the concentration of administered C1q, the site of injection and the experimenter being constant, we observed considerable interindividual differences in the recovery rate of C1q-reconstituted mice, potentially due to subclinical differences in the intraperitoneal injection site. As a consequence, for the analysis of tail bleeding experiments following C1q reconstitution, only mice in which at least 5 μg/ml of C1q plasma concentration could be achieved were included in the analysis.

### Lectin Pathway Activity

Mice were injected with either saline or C1q as described previously and serum was obtained 2 h later. Serum samples were analyzed for the degree of lectin pathway activity by a mouse lectin pathway assay ELISA kit (Hycult). Lectin pathway activity was calculated expressing the obtained OD values of samples in percent compared to the provided positive control of the kit.

### Statistical Analysis

Data are expressed as median ± interquartile range (IQR), if not stated otherwise. Mann–Whitney test was used to compare two sets of non-parametric, unpaired data. Correlations were estimated by Spearman's rank correlation coefficient. Data were analyzed with a statistical package program (GraphPad Prism 8, La Jolla, CA, USA). Differences were considered statistically significant when the *p*-value was <0.05.

## Results

### Complete Blood Counts and vWF Concentrations of C1q-Deficient vs. WT Mice

**Figure 1:**
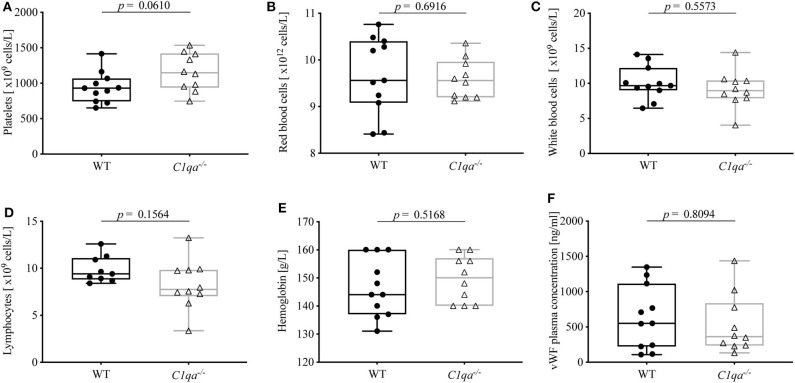
Complete blood counts and vWF levels of C1q-deficient vs. WT mice. EDTA-anticoagulated whole blood of C1q-deficient and WT mice was obtained and quantified by flow cytometry for numbers of **(A)** platelets, **(B)** red blood cells, **(C)** white blood cells, **(D)** lymphocytes, and **(E)** amount of hemoglobin. **(F)** From citrated blood, vWF plasma concentration was quantified using ELISA. Horizontal lines in the box plots denote median while the boxes indicate interquartile range and whiskers minimum and maximum values. Data points represent individual mice, **(A–F)** 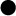: *n* = 11, △: *n* =10; **(D)** 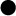: *n* = 9, △: *n* = 10 (Mann–Whitney test; ns, not significant).

In order to exclude confounders that can influence bleeding behavior, we first assessed hematologic parameters of the two strains. In detail, blood of WT mice and C1q-deficient mice was analyzed for blood counts (red blood cells, white blood cells, platelets, lymphocytes and hemoglobin) by flow cytometry, and vWF levels were quantified by ELISA. There was no statistically significant difference between WT and C1q-deficient mice for all of these parameters (Figure 1).

### Prothrombin Time and Activated Partial Thromboplastin Time of C1q-Deficient vs. WT Mice

**Figure 2:**
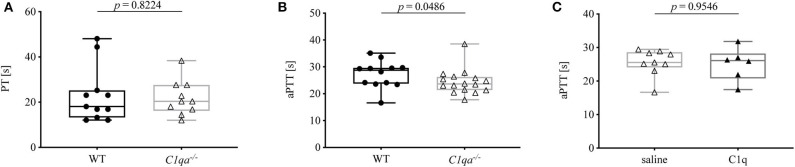
Prothrombin time and activated partial thromboplastin time of C1q-deficient vs. WT mice. **(A)** Citrated whole blood of WT and C1q-deficient mice was analyzed for prothrombin time. **(B,C)** Citrated blood plasma of **(B)** WT and C1q-deficient mice and of **(C)** saline injected and C1q injected C1q-deficient mice was analyzed for activated partial thromboplastin time. Horizontal lines in the box plots denote median while the boxes indicate interquartile range and whiskers minimum and maximum values. Data points represent individual mice, **(A)** *n* = 10 for each group; **(B)** 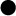: *n* = 12, △: *n* = 16; **(C)** △: *n* = 9, ▴: *n* = 6 (Mann–Whitney test).

Secondary hemostasis can be assessed by two different *in vitro* global coagulation tests. The PT provides information on the extrinsic pathway whereas the aPTT assesses the intrinsic pathway. In this way, abnormalities in coagulation factors of either pathway can be determined (18). The PT of C1q-deficient mice did not differ significantly from WT mice (Figure 2A). Even though the aPTT was shorter in C1q-deficient than in WT mice (median aPTT (IQR) of C1q-deficient mice: 23.63 s (21.35–26.25 s) vs. WT mice: 28.75 s (23.73–29.65 s), *p* = 0.0486) (Figure 2B), administration of C1q to C1q-deficient mice did not result in a prolonged aPTT compared to saline injected C1q-deficient mice (median aPTT (IQR) of C1q injected mice: 26.10 s (20.79–28.20 s) vs. saline injected mice: 25.50 s (24.00–28.58 s), *p* = 0.9546) 2h after injection (Figure 2C).

### Platelet Aggregation of C1q-Deficient vs. WT Mice

**Figure 3:**
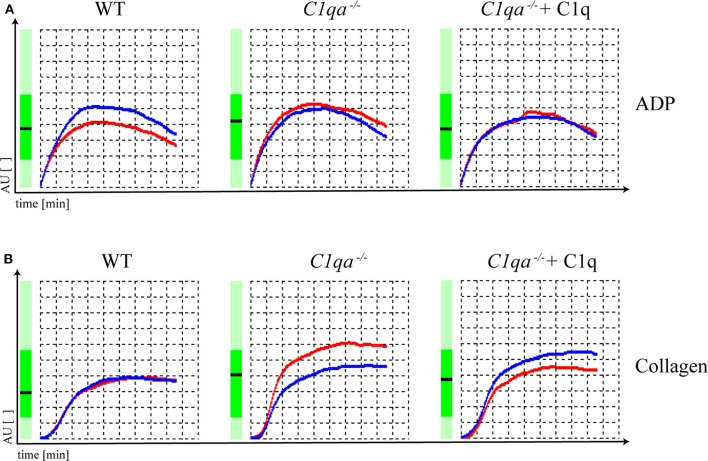
Platelet function of C1q-deficient vs. WT mice. Hirudin-anticoagulated whole blood was obtained for functional platelet aggregation tests using **(A)** ADP (15 μM) and **(B)** collagen (10 μg/ml) as inducers of platelet aggregation. Platelet aggregation curve of saline injected WT mice, saline injected C1q-deficient mice and C1q injected C1q-deficient mice are shown. Electrical impedance is expressed as aggregation units (AU) and plotted over time [min]. Representative data of one out of two independent experiments are shown. For each experiment, blood from four mice per group was pooled.

Platelet function can be assessed by various methods. An elegant way is the impedance whole blood aggregometry. This method allows platelets to adhere to a solid surface, which resembles the physiological function of platelets *in vivo*. Adhesion of platelets to fixed electrodes results in an increase of electrical impedance that is transformed to arbitrary aggregation units (AU) and plotted against time (19), from where the area under the curve (AUC) can be calculated (10 AU ^*^ min = AUC [U]). In our study, we obtained hirudin-anticoagulated whole blood and induced platelet aggregation with ADP and collagen. The AUC range in which human platelets are considered to be responsive is from 321 to 1059 U when platelet aggregation is induced by ADP and from 242 to 1019 U when platelet aggregation is induced by collagen (Multiplate® analyzer, validated for hospital use). The AUC for ADP-induced platelet aggregation was 677 U for saline injected WT mice, 756 U for saline injected C1q-deficient mice and 669 U for C1q injected C1q-deficient mice 2 h after injection. For collagen-induced platelet aggregation, an AUC of 533 U for saline-injected WT mice, 741 U for saline injected C1q-deficient mice and 677 U for C1q injected C1q-deficient mice was observed 2 h after injection (Figure 3). Based on criteria used in clinics, all AUCs indicate the functional responsiveness of platelets that were obtained from all three groups.

### Complement Activation Following C1q Reconstitution

Since C1q is commonly known for its complement activating effect and complement activation can in turn activate hemostasis, we addressed whether the injection of human C1q triggers complement activation in mice.

Importantly, C1q-deficient mice have been described to have no impairment of complement activation, neither via the lectin pathway nor via the alternative pathway (20)Since the classical and lectin pathways, after initiation through their respective recognition molecules, proceed through the same complement components, we assessed the complement activation by measuring the lectin pathway activity. Activity of the lectin pathway of C1q-deficient mice 2 h after reconstitution with C1q was comparable to the activity of non-reconstituted control mice [median lectin pathway activity (IQR) of C1q-injected mice: 29.90 % (22.74–32.55 %) vs. saline-injected mice 27.15 % (21.10–36.38 %), *p* = 0.5476] (Supplementary Figure 3A). Moreover, there was no correlation of the lectin pathway activity with the achieved C1q concentrations after reconstitution (Spearman *r*= −0.1000, *p* = 0.9500) (Supplementary Figure 3B).

### C1q-Deficient Mice Show Enhanced Bleeding Diathesis

**Figure 4:**
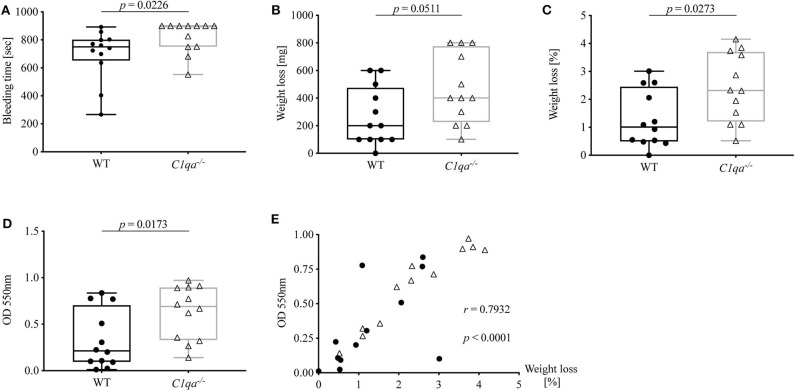
Bleeding tendency of C1q-deficient vs. WT mice. Tail bleeding assay was performed and bleeding tendency of C1q-deficient and WT mice assessed by **(A)** bleeding time, **(B)** weight loss, **(C)** relative weight loss normalized to the total body weight and **(D)** OD of obtained blood-PBS solution. **(E)** Correlation between OD and relative weight loss is depicted. Horizontal lines in the box plots denote median while the boxes indicate interquartile range and whiskers minimum and maximum values. Data points represent individual mice, *n* = 12 per group (Mann–Whitney test; *r*, Spearman's rank correlation coefficient).

Accumulating evidence highlights the cross-talk between complement and coagulation (21, 22). Previously, our group described the occurrence of C1q-vWF complexes *in vitro* as well as *ex vivo*. Hence, we wanted to investigate whether C1q deficiency also impacts on hemostasis. For this, we conducted a tail bleeding assay and found that the bleeding time of C1q-deficient mice was significantly prolonged compared to WT mice [median bleeding time (IQR) for C1q-deficient mice: 900 s (750.5–900.0 s) vs. WT mice 750.5 s (651.8–802.0 s), *p* = 0.0226] (Figure 4A). Noteworthy, 900 s were equivalent to the upper time limit of the experimental procedure. Moreover, during the tail bleeding assay C1q-deficient mice lost twice the amount of blood [median weight loss (IQR) in mg of C1q-deficient mice 400 mg (225–775 mg) vs. WT mice: 200 mg (100–475 mg), *p* = 0.0511] (Figure 4B) and 2.3-fold the amount when normalized to their body weight [median weight loss (IQR) in % of C1q-deficient mice: 2.32% (1.21–3.70%) vs. WT mice: 1.01% (0.49–2.46 %), *p* = 0.0273] (Figure 4C) compared to WT mice. The loss of blood could be confirmed when measuring the optical density of the resulting blood-PBS solution. The OD of the obtained solution from C1q-deficient mice showed a 3.2-fold increase compared to WT mice [median OD at 550 nm of C1q-deficient mice: 0.69 (0.33–0.90) vs. WT mice: 0.21 (0.09–0.70), *p* = 0.0173] (Figure 4D). In addition, there was a positive correlation between the OD and the relative weight loss (Spearman *r* = 0.7932, *p* < 0.0001) (Figure 4E).

To summarize, C1q-deficient mice exhibit an enhanced bleeding diathesis compared to WT mice.

### Reconstitution of C1q-Deficient Mice With Purified C1q

To confirm that the prolonged bleeding time in C1q-deficient mice can be attributed to the lack of C1q, we next performed tail bleeding experiments with or without reconstitution with C1q. First, we quantified the C1q serum concentration of WT and C1q-deficient mice. The median C1q serum concentration of WT mice was 192.4 μg/ml, whereas the C1q serum concentration of C1q-deficient mice was below the lower limit of detection (Supplementay Figure 2A).

Second, we then investigated the kinetics of recovery for the reconstitution of C1q-deficient mice with human C1q. Injecting the highest administrable dose of C1q, a partial reconstitution, equivalent to ~30% of the concentration of murine C1q in WT mice, was achieved with its maximum at 58.75 μg/mL after 2 h (Supplementary Figure 2B). Hence, for C1q reconstitution experiments, C1q-deficient mice were *i.p*. injected with either human C1q or 0.9% saline and experiments carried out after 2 h.

### C1q Reconstituted C1q-Deficient Mice Show a Reduced Bleeding Tendency

In order to investigate whether the observed prolonged bleeding tendency of C1q-deficient mice could be rescued, and thus can be attributed to the lack of C1q, we reconstituted C1q-deficient mice with human C1q or injected 0.9% saline as control and performed tail bleeding experiments in the same manner as described previously. Subsequently, C1q plasma concentrations were analyzed to evaluate the degree of C1q reconstitution.

**Figure 5:**
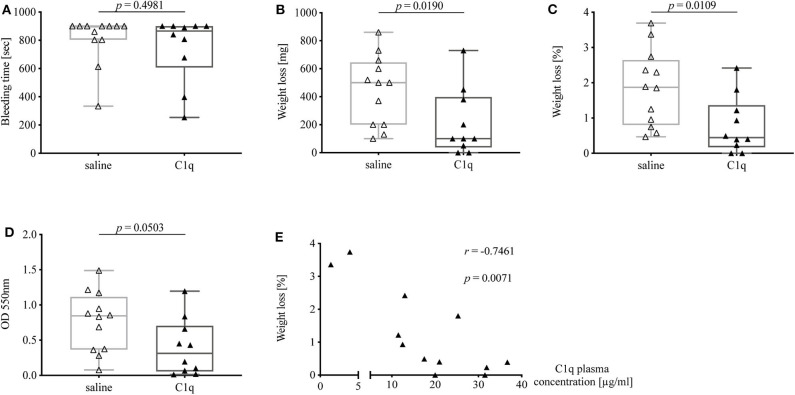
Bleeding tendency of C1q-deficient mice with or without reconstitution with C1q. Tail bleeding assay was performed and bleeding tendency in C1q-deficient mice injected *i.p*. with either C1q or 0.9% saline 2 h prior to assessment of **(A)** bleeding time, **(B)** weight loss, **(C)** relative weight loss normalized to the total body weight, and **(D)** OD of obtained blood-PBS solution. **(E)** Correlation between relative weight loss and achieved C1q plasma concentration in C1q-reconstituted C1q-deficient mice is shown. Horizontal lines in the box plots denote median while the boxes indicate interquartile range and whiskers minimum and maximum values. Data points represent individual mice, △: *n* = 12, ▴: *n* = 10 (Mann–Whitney test; *r*, Spearman's rank correlation coefficient).

The bleeding time of C1q-deficient mice reconstituted with C1q was slightly shorter than in non-reconstituted control mice [median bleeding time (IQR) of C1q-injected mice: 865 s (607.0–900.0 s) vs. saline-injected mice 900 s (804–900 s), *p* = 0.4981] (Figure 5A). Strikingly, mice that were reconstituted with C1q lost one fifth of the amount of blood of mice that were injected with saline instead [median weight loss (IQR) in mg of C1q-injected mice: 100 mg (37.5–397.5 mg) vs. saline-injected mice 500 mg (200–645 mg), *p* = 0.0190] (Figure 5B) and 4.2-fold less amount of blood when normalized to their body weight [median weight loss (IQR) in % of C1q injected mice: 0.45% (0.17–1.37 %) vs. saline-injected mice: 1.87% (0.80–2.65 %), *p* = 0.0109] (Figure 5C). Moreover, the OD of obtained blood solution from C1q reconstituted mice showed a 2.7-fold decrease compared to control mice [median OD at 550 nm of C1q-injected mice: 0.31 (0.06–0.70) vs. saline-injected mice: 0.85 (0.37–1.12), *p* = 0.0503] (Figure 5D). Most strikingly, the relative weight loss of C1q reconstituted mice during tail bleeding experiment correlated negatively with the achieved C1q concentrations after reconstitution (Spearman *r* = −0.7461, *p* = 0.0071) (Figure 5E).

Notably, when comparing bleeding tendency of C1q-reconstituted with WT mice, bleeding time of C1q-reconstituted mice approached the times as observed in WT mice, while the absolute as well as relative weight loss was nearly identical between C1q-reconstituted mice and WT mice suggesting that the lack of C1q was fully responsible for differences observed between C1q-deficient and WT mice.

### Discussion

The cross-talk between the complement system and the coagulation system is becoming ever more apparent with many of the interactions still not being fully understood. Our previous research has shown that a complex of C1q and vWF is able to recruit human platelets indicating that C1q has the potential to mediate hemostasis (14). Therefore, we sought to examine whether C1q can impact on blood coagulability. For this purpose, we compared C1q-deficient mice to WT mice with regard to qualitative and quantitative analyses of parameters of primary and secondary hemostasis. Our study demonstrates that C1q-deficient mice exhibit prolonged bleeding and increased blood loss compared to WT mice during tail bleeding experiments. Performing reconstitution experiments with human purified C1q, the altered bleeding behavior of C1q-deficient mice could be reversed and correlated with the achieved C1q concentration in those mice. These findings highlight that C1q is directly involved in thrombus formation during coagulation.

Similar to humans, clinical conditions characterized by a decline in blood platelets (thrombocytopenia) or vWF levels (von Willebrand factor disease) can lead to dysfunctional hemostasis and thrombosis in mice (23, 24). In order to exclude such quantitative differences, we compared complete blood counts and vWF plasma concentrations of C1q-deficient and WT mice. As expected, counts of platelets, red blood cells, white blood cells, and lymphocytes as well as levels of hemoglobin and vWF did not differ significantly (Figure 1). Remarkably, the trend of higher platelet counts in C1q-deficient mice, which might rather imply increased coagulability in these mice, could be due to higher platelet consumption during blood collection in WT mice.

PT and aPTT are employed in the clinics to monitor clotting time, e.g., for assessment of anticoagulant therapy, and to diagnose bleeding disorders, e.g., due to deficiencies in coagulation factors. While PT and aPTT both give insight into a functional common pathway involving factors X, V, and II, the PT measures the integrity of the extrinsic pathway involving TF and factor VII, whereas the aPTT provides information on the intrinsic pathway involving factors XII, XI, IX, and VIII. While prolonged PT and aPTT usually indicate impaired secondary hemostasis, shortened PT or aPTT due to abnormal levels of coagulation factors, such as factor XII, does not necessarily translate into coagulation disorders (18, 25). Since no defect in coagulation factors has been described for C1q-deficient mice, we expected the two tested mouse strains to show no significant differences in PT and aPTT. While PT was comparable, unexpectedly, the activated partial thromboplastin time was significantly shorter in C1q-deficient mice compared to WT mice (Figure 2). This finding would be indicative of an increased coagulability of C1q-deficient mice, and thus oppose the enhanced bleeding behavior. Since previous research has demonstrated that C1q exerts an inhibitory effect on factor XII under physiological conditions, absence of C1q might result in an overly active factor XII, hereby shortening the aPTT (9). Another explanation for the shorter aPTT might be an indirect effect of the formation of C1q-vWF complexes. Physiologically, vWF is necessary for the transport of factor VIII to the site of secondary hemostasis on activated platelets. Thus, deficiency in C1q might result in its omission as a binding partner and hence in a shifted balance of vWF function toward carrying factor VIII, hereby enhancing factor VIII function and leading to a shortened aPTT. In line with this hypothesis, C1q reconstitution in C1q-deficient mice did not lead to an immediate prolongation of the aPTT (Figure 2C).

Next to vWF dysfunction or quantitative platelet defects, qualitative platelet defects, such as dysfunctional platelet membrane receptors, can be causative for bleeding disorders (26, 27). Several studies investigating the effect of C1q on collagen-induced platelet aggregation have shown controversial findings, either weakening or potentiating platelet aggregation (6, 7, 28, 29). Peerschke and Ghebrehiwet have repeatedly studied C1q receptors on platelets as well as the consequences of C1q stimulation on platelet activation, concluding that C1q binding to several platelet receptors leads to platelet activation via P-selectin induction and thus increases procoagulant activity (5, 30, 31).

However, Kölm et al. have not observed binding of platelets to C1q, whereas platelets are enabled to adhere to the C1q-vWF complex in an *in vitro* flow-chamber model (14). Additionally, interaction between C1q and endothelial cells has been demonstrated. C1q has been shown to induce endothelial cell adhesion and spreading, which in turn leads to a prothrombotic phenotype of these cells (32, 33).

In our study, analyzing platelet aggregation induced by collagen and ADP, platelets of WT as well as C1q-deficient mice were found to be responsive and there were no functional differences in platelets of C1q-deficient mice with or without C1q reconstitution (Figure 3). Additionally, the augmenting effect of C1q on primary hemostasis appeared to be independent of downstream complement activation as we observed similar total lectin pathway activity in C1q and saline injected C1q-deficient mice suggesting that complement activation after reconstitution with C1q was low during the experimental procedure (Supplementary Figure 3).

Taken together, we demonstrate that C1q-deficient mice exhibit augmented bleeding (Figure 4) that can be partially reversed by reconstitution with C1q (Figure 5).

These results suggest that C1q is directly involved in the maintenance of hemostasis. Conceivably, C1q might represent a binding partner for vWF not only *in vitro* but also *in vivo*. Consequently, the lack of C1q potentially results in reduced vWF binding followed by diminished platelet aggregation and subsequent prolongation of bleeding.

However, considering the substantial body of evidence from C1q-cell and C1q-surface molecule interaction studies, we cannot exclude an (maybe additional) interplay between (i) C1q and platelets directly, (ii) C1q and endothelial cells, or (iii) C1q and negatively charged surface molecules, or (iv) a still unknown interplay between C1q and another component of coagulation to participate in hemostasis.

Therefore, a limitation of our study is that, although we propose the C1q-vWF interaction to be responsible for the altered bleeding tendency, the precise mechanism remains to be clarified. Furthermore, the impact of an evolving hemorrhagic shock during our tail bleeding assay cannot be fully excluded. Interpreting findings of studies that investigate the effect of severe blood loss on activation of complement and coagulation (exclusively based on blood loss without the concomitant presence of sepsis or polytrauma) (34, 35), we conclude that even though our mice experience significant hemorrhage during tail bleeding assays, subsequent complement activation is less likely to be a confounder in our results, whereas activation of coagulation seems to be plausible. Last, our study is limited by the use of human instead of murine C1q for reconstitution experiments. Nevertheless, a 76% sequence homology on DNA level and a 72% homology on protein level exists between human and murine C1q (36). Although human C1q levels in C1q-reconstituted mice remained rather lower than murine C1q levels in WT mice (Supplementary Figure 2B), we observed significant differences in blood loss between C1q-reconstitued and non-reconstituted mice (Figures 5B,C), indicating that human C1q is capable of mimicking functions of murine C1q.

The clinical relevance of our observations remains purely hypothetical. A well-balanced complement system is crucial to protect against pathogens and fight against infections. Considering the hemostatic effects of bound C1q as observed in our study, it would be plausible that large amounts of more systemically deposited C1q–as occurring in certain diseases–also have procoagulant effects. In line with this hypothesis, conditions where an overly active complement system results in high consumption and deposition of complement C1q, such as in bacterial sepsis and SLE, frequently lead to severe thrombotic complications at the same time. Regarding sepsis, it has been demonstrated that gram-positive bacteria-induced sepsis is accompanied by significant C1q consumption (37). Additionally, C1q has been described to bind to gram-positive and gram-negative bacteria (38).

However, independent of effects by C1q alone, downstream activation of complement and generation of inflammatory mediators, such as C3a and C5a, has been shown to stimulate circulating neutrophils and endothelial cells and subsequently upregulates TF expression on these cells (39, 40). The enhanced TF expression fuels activation of the contact system of hemostasis and thus leads to increased thrombogenicity. This in turn can result in sepsis-associated coagulopathies that have been suggested to negatively affect the outcome by increasing mortality in these patients (41). In general, inflammation is considered a predisposing factor for thrombosis.

Another example of such cross-talk is SLE, the prototype of systemic autoimmune diseases. SLE is commonly characterized by low plasma levels of C1q due to high consumption and deposition (42, 43). Strikingly, SLE patients have been described to have a higher risk of thrombotic complications, which cannot be sufficiently explained by traditional risk factors (44, 45). Those thrombotic complications include platelet hyperfunction (46), thrombotic microangiopathies (TMA) (47), venous thromboembolism (VTE) (48) and atherosclerosis (49). Moreover, it has been shown that C1q and other complement components are deposited on platelets of SLE patients and are associated with venous as well as arterial thrombotic events in those patients (50–52).

Apart from bacterial sepsis and SLE, C1q has also been found to be present in high concentrations at sites of atherosclerotic, inflammatory and vascular lesions, and vice versa high concentrations of C1q in these conditions have been postulated to be a driver for inflammation and thrombosis (53–55). Therefore, future work is warranted to elucidate the clinical relevance of C1q for hemostasis in humans.

In conclusion, our study provides evidence that C1q has a physiological role in hemostasis *in vivo* by promoting arrest of bleeding. With regard to disease, an excess of deposited C1q might be a cause of thrombotic complications in inflammatory diseases such as bacterial sepsis and SLE.

## Data Availability Statement

All datasets generated for this study are included in the article/Supplementary Material.

## Ethics Statement

The animal study was reviewed and approved by Cantonal Commission for Animal Experiments, and the Federal Food Safety and Veterinary Office (license number 2898/28447).

## Author Contributions

CD, RK, DT, and MT designed the study. CD, KC, and ET performed experiments. CD collected, analyzed data and wrote the manuscript. MT supervised the study. All authors critically revised the manuscript. All authors contributed to the article and approved the submitted version.

## Conflict of Interest

The authors declare that the research was conducted in the absence of any commercial or financial relationships that could be construed as a potential conflict of interest.

## Supplementary Material

Supplementary Figure 1 Experimental set-up of tail bleeding assay. **(A)** Mice are anesthetized and placed on individual platforms. Ten millimeter of the distal tail is cut and tails immersed in 37°C prewarmed PBS solution. Time of bleeding is observed for 15 min. **(B)** Blood-PBS solution obtained from tail bleeding of individual mice is shown. **(C)** Blood-PBS solution is pipetted into a 96 well-plate for analysis of OD.

Supplementary Figure 2 Baseline serum C1q concentrations and concentrations of serum C1q in reconstituted mice over time. **(A)** Baseline C1q serum concentration of WT and C1q-deficient mice was quantified by ELISA. **(B)** C1q-deficient mice were *i.p*. injected with purified human C1q and C1q concentration was quantified from serum obtained after 30 min, 1, 2, and 8 h after injection. Columns denote median while errors bars indicate IQR. Data points represent individual mice, **(A)** λ: *n* = 7, ρ: *n* = 5; **(B)** *n* = 2.

Supplementary Figure 3 Lectin pathway activity of C1q-deficient mice with or without reconstitution with C1q. **(A)** Lectin pathway (LP) activity of saline injected and C1q injected C1q-deficient mice was quantified by ELISA and expressed in percent relative to the positive control (ctrl). **(B)** Correlation between LP activity and achieved C1q serum concentration in C1q-reconstituted C1q-deficient mice is shown. Horizontal lines in the box plots denote median while the boxes indicate interquartile range and whiskers minimum and maximum values. Data points represent individual mice, *n* = 5 for each group (Mann–Whitney test; *r*, Spearman's rank correlation coefficient).
