# Supplementary figures and images for: Complement C1q Enhances Primary Hemostasis
Source: Front Immunol. 2020 Jul 16;11:1522. doi: 10.3389/fimmu.2020.01522 (PMC7381122; doi:10.3389/fimmu.2020.01522)

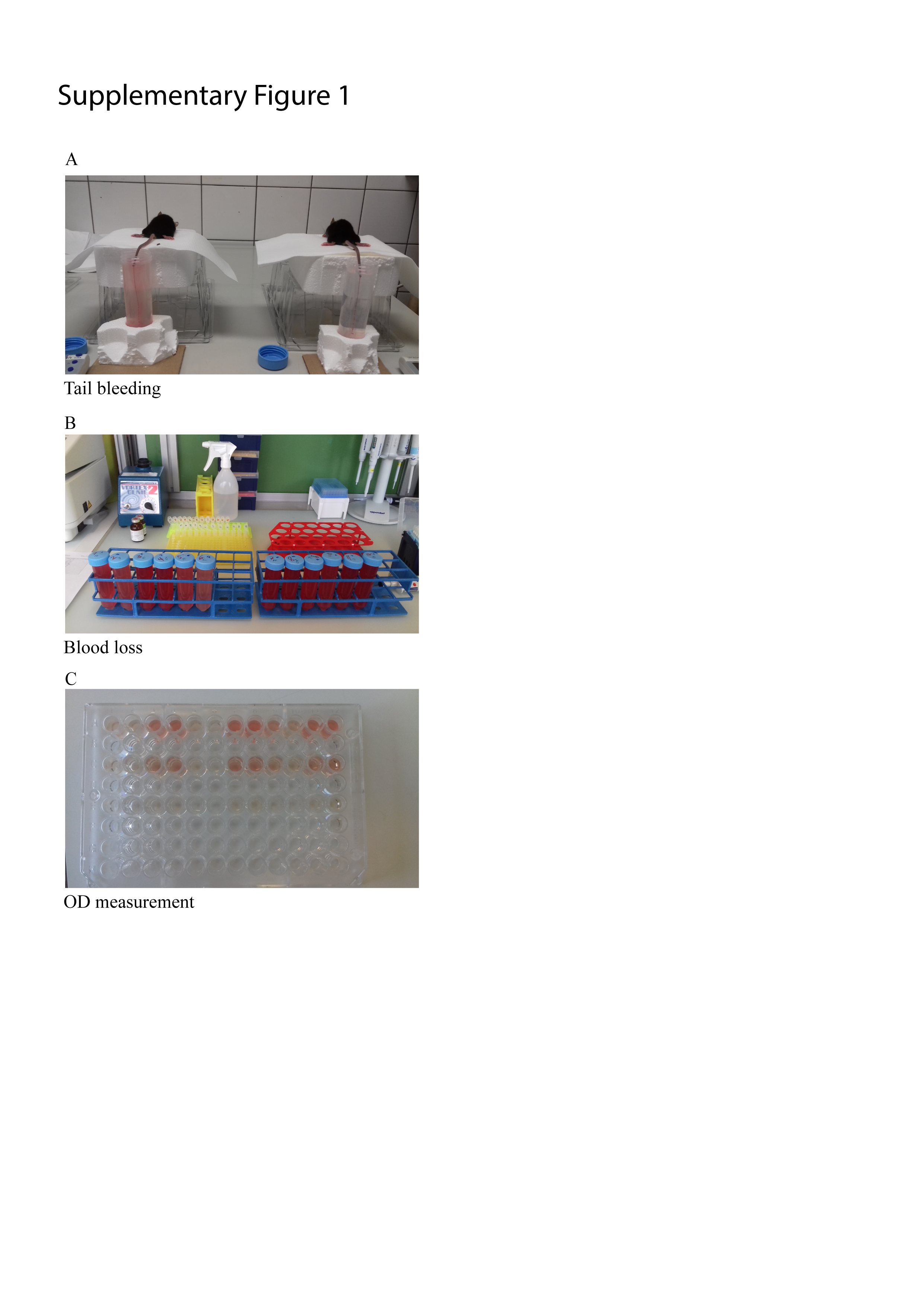

Supplement: Supplementary Figure 1 — Experimental set-up of tail bleeding assay. (A) Mice are anesthetized and placed on individual platforms. Ten millimeter of the distal tail is cut and tails immersed in 37°C prewarmed PBS solution. Time of bleeding is observed for 15 min. (B) Blood-PBS solution obtained from tail bleeding of individual mice is shown. (C) Blood-PBS solution is pipetted into a 96 well-plate for analysis of OD. [file Image_1.TIF]

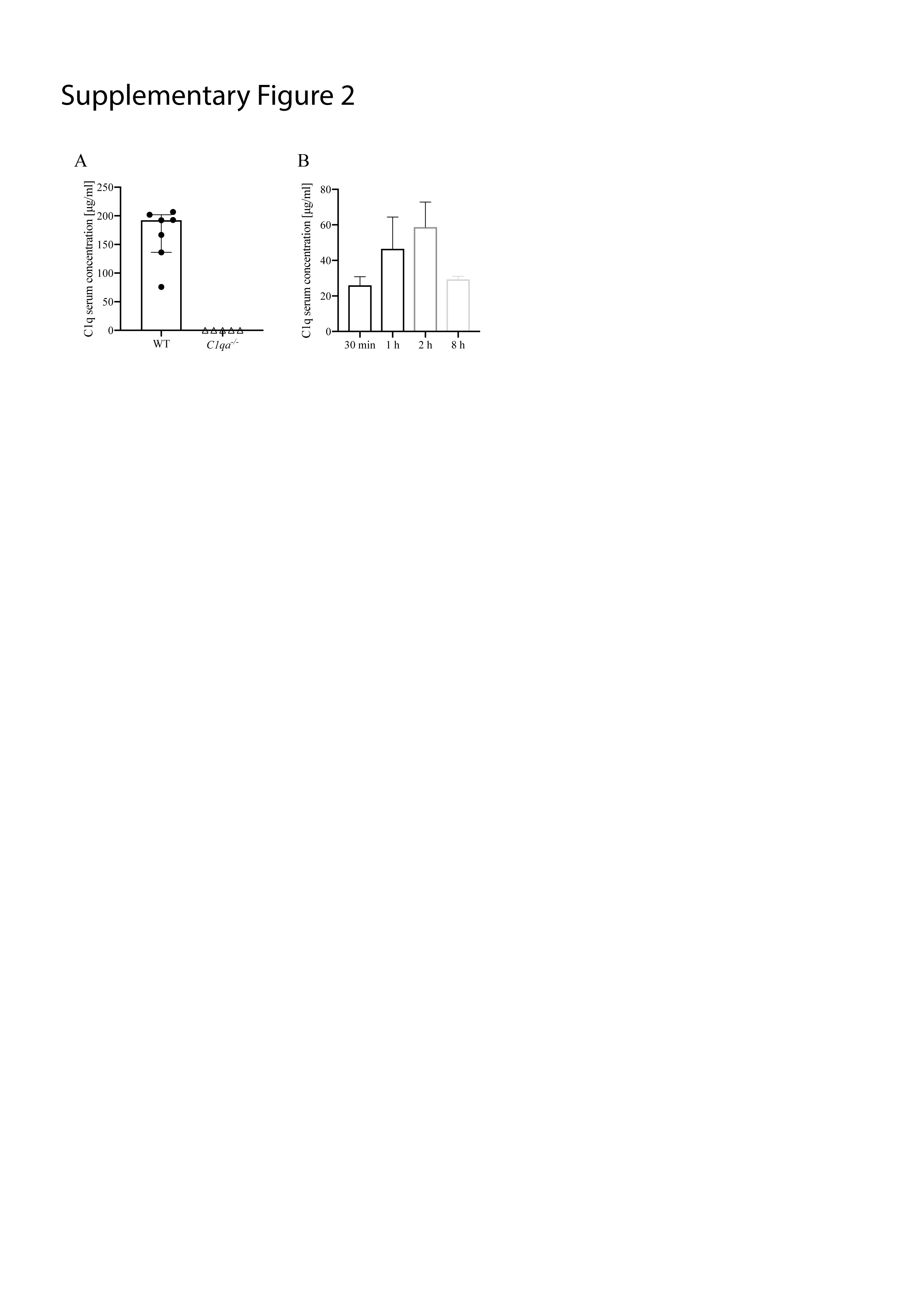

Supplement: Supplementary Figure 2 — Baseline serum C1q concentrations and concentrations of serum C1q in reconstituted mice over time. (A) Baseline C1q serum concentration of WT and C1q-deficient mice was quantified by ELISA. (B) C1q-deficient mice were i.p. injected with purified human C1q and C1q concentration was quantified from serum obtained after 30 min, 1, 2, and 8 h after injection. Columns denote median while errors bars indicate IQR. Data points represent individual mice, (A) λ: n = 7, ρ: n = 5; (B) n = 2. [file Image_2.TIF]

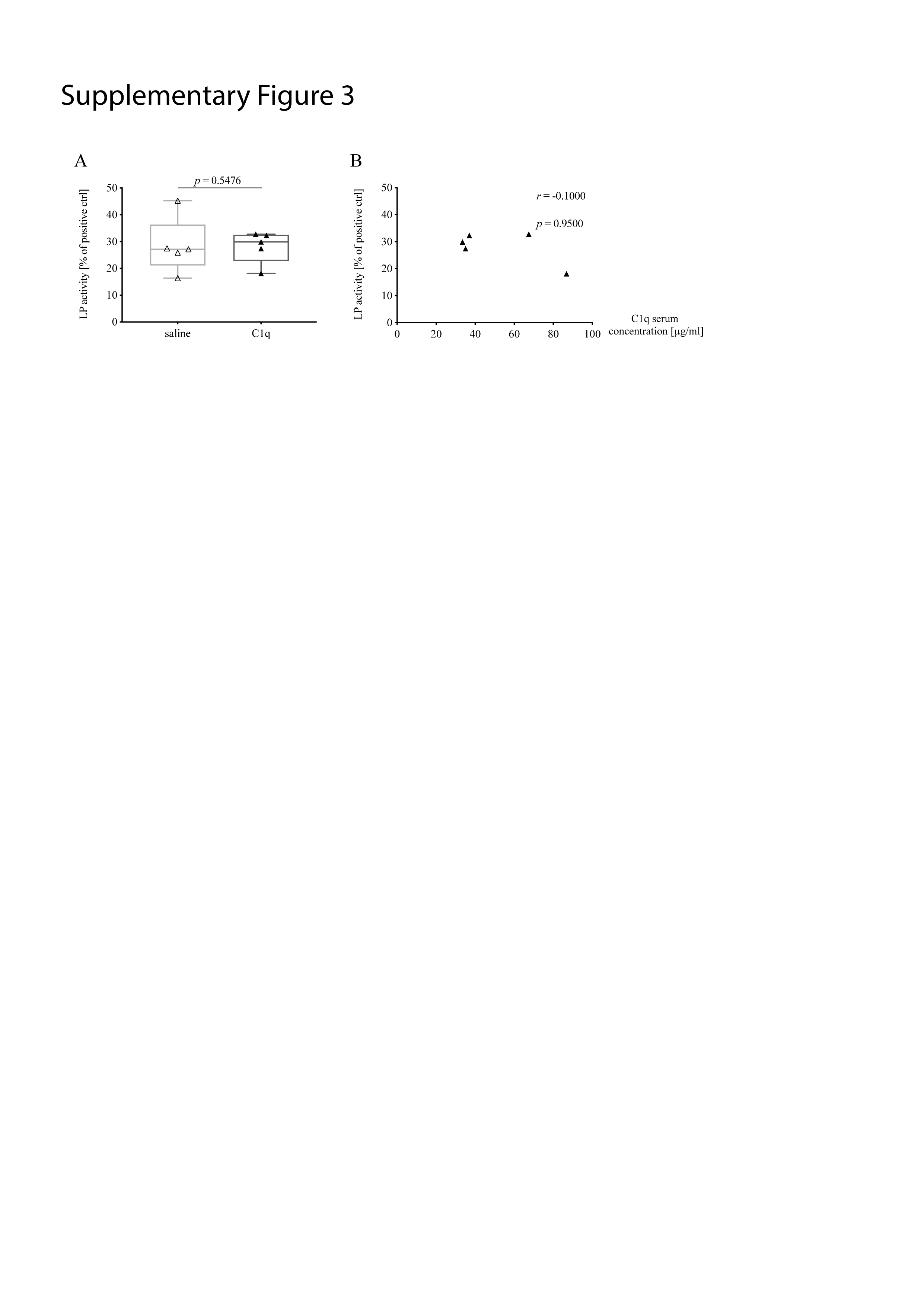

Supplement: Supplementary Figure 3 — Lectin pathway activity of C1q-deficient mice with or without reconstitution with C1q. (A) Lectin pathway (LP) activity of saline injected and C1q injected C1q-deficient mice was quantified by ELISA and expressed in percent relative to the positive control (ctrl). (B) Correlation between LP activity and achieved C1q serum concentration in C1q-reconstituted C1q-deficient mice is shown. Horizontal lines in the box plots denote median while the boxes indicate interquartile range and whiskers minimum and maximum values. Data points represent individual mice, n = 5 for each group (Mann–Whitney test; r, Spearman's rank correlation coefficient). [file Image_3.TIF]
